# Palaeotoxicity: reconstructing the risk of multiple sedimentary pollutants to freshwater organisms

**DOI:** 10.1007/s10653-018-0080-5

**Published:** 2018-03-02

**Authors:** Neil L. Rose, Simon D. Turner, Handong Yang, Congqiao Yang, Charlotte Hall, Stuart Harrad

**Affiliations:** 1 Environmental Change Research Centre, Department of Geography, University College London, Gower St, London, WC1E 6BT UK; 2 Present Address: Department of Earth Sciences, University of Toronto, Toronto, ON M5S 3B1 Canada; 3 School of Geography, Earth and Environmental Sciences, University of Birmingham, Birmingham, B15 2TT UK

**Keywords:** Lake sediments, Palaeotoxicity, Persistent organic pollutants, Risk, Sediment-dwelling biota, Trace metals

## Abstract

‘Real-world’ contaminant exposure of sediment-dwelling biota is typically long-term, low-level and to multiple pollutants. However, sediment quality guidelines, designed to protect these organisms, relate only to single contaminants. This study uses radiometrically dated sediment cores from 7 English lakes with varying contamination histories to reconstruct temporal changes in likely risk to biota (herein termed ‘palaeotoxicity’). The Probable Effects Concentration Quotient (PEC-Q) approach was used to combine sediment concentrations from multiple contaminants (trace metals; PCBs; PBDEs) to determine risk allocated to metals and persistent organic pollutants (POPs) separately as well as combined (PEC-Q Mean-All). Urban-influenced lakes were considerably more contaminated, exceeding PEC-Q thresholds of 0.5 and 2.0 over long durations (some since the nineteenth century). This has been mainly due to metals (principally lead) and by factors of up to 10 for individual metals and by > 2 for PEC-Q Mean-Metals. In 6 out of 7 lakes, considerable reductions in risk associated with trace metals are observed since emissions reductions in the 1970s. However, at all lakes, PEC-Q Mean-POPs has increased sharply since the 1950s and at 5 out of 7 lakes now exceeds PEC-Q Mean-Metals. These organic pollutants are therefore now the dominant driver behind elevated contaminant risk to sediment-dwelling biota and recent temporal trends in PEC-Q Mean-All remain above threshold values as a result. Finally, PEC-Q Mean-All values were compared to standard biological toxicity tests for surface sediments at each site. While chironomid growth and daphniid reproduction were significantly reduced compared to controls at 5 out of 7, and all lakes, respectively, the scale of these reductions showed only limited quantitative agreement with predicted risk.

**Electronic supplementary material:**

The online version of this article (10.1007/s10653-018-0080-5) contains supplementary material, which is available to authorized users.

## Introduction

Lakes are located at the bottom of hydrological watersheds and therefore act as natural sinks for contamination deposited from the atmosphere, transported from upstream sources or remobilised from catchments (Bogdal et al. 2010; Rose et al. 2012). Many pollutants entering lakes adsorb to organic and inorganic particulates and so lake sediments tend to act as long-term accumulators. As a result, the real-world exposure of lake sediment-dwelling biota is typically to a mixture of contaminants, usually at low-level concentrations and over long periods of time (e.g. the life time of the organism) (Leung et al. 2005; Beyer et al. 2014) although studies on the effects of multiple pollutants are rare (Norwood et al. 2003).

In order to determine the scale of contamination in freshwater sediments and the probability of risk they pose to sediment-dwelling organisms, Sediment Quality Guidelines (SQGs) have been developed by a broad range of agencies (e.g. MacDonald et al. 2000; Ingersoll et al. 2001). These generally comprise two levels of probability for harm: Threshold Effects Concentrations (TECs) defined as sediment contaminant concentrations below which harmful effects on sediment-dwelling organisms would not be expected, and Probable Effects Concentrations (PECs) above which harmful effects would be expected to occur frequently due to that pollutant alone (MacDonald et al. 2000). Sediment Quality Guidelines, therefore, only consider the probability of harm from individual pollutants in isolation. However, in the field, any observed detrimental impact due to a pollutant is only ever likely to be a response due to that chemical in the presence of others (Leung et al. 2005) and hence predictions of an organism’s exposure are usually underestimated (Walter et al. 2002).

The lake sediment record provides a natural archive of contaminant inputs to lakes over decades and centuries and robust radiometric dating can place this in a highly resolved temporal context. The sediment record can therefore provide temporal information on pollutant inputs in the absence of long-term monitoring data; a means to determine the scale of contamination over a natural baseline; and site-specific, empirical, pre-contamination ‘reference conditions’ against which to measure restoration targets and mitigation success. Lake sediment records have been widely used to determine temporal trends of a broad range of pollutants including trace metals (Boyle et al. 1998; Yang et al. 2002, 2010); persistent organic pollutants (Rose et al. 2001; Yang et al. 2016), pharmaceuticals (Kerrigan et al. 2018) and fly-ash particles (Rose et al. 2012; Rose 2015). However, despite a number of approaches employed to determine the effects of sediment contaminant mixtures to aquatic biota (for example Probable Effects Concentration Quotients (PEC-Qs), Ingersoll et al. 2001; toxicity units (TUs), Lahr et al. 2003; Hazard Quotients (HQs), Feng et al. 2011; geoaccumulation indices (I_geo_), Haris et al. 2017), these have mainly been applied to surface sediments. Only rarely have these techniques been used with lake sediment records to determine historic toxic effects, even though this would allow contemporary status to be placed in an historical perspective such that directions of change (deterioration; improvement) could be determined as well as, perhaps more importantly, rates of change. Cook et al. (2003) reconstructed the impact of a combination of dioxins, furans (PCDD/Fs) and polychlorinated biphenyls (PCBs) on Lake trout (*Salvelinus namaycush*) eggs from a Lake Ontario sediment core and showed 100% mortality over a 25-year period from c.1950 to the mid-1970s. These data showed how toxic effects rapidly declined to contemporary sub-lethal effects in recent decades, in agreement with observed data. Hall (2013) assessed the historical effects of a combination of trace metals in a number of urban ponds in London using the PEC-Q approach and found an enhanced probability of detrimental biological impact over much of the twentieth century.

Here, we provide, to our knowledge, the first historical reconstruction of toxicity risk (which we term ‘palaeotoxicity’) using both trace metals and persistent organic pollutants (POPs). We use chronologically constrained lake sediment data for these pollutants, from both urban and rural lakes across the UK, to determine (1) the historical trends in likely detrimental impacts to biota, (2) which contaminants are the most probable drivers behind these impacts, and (3) how these predicted results compare with standard biological tests for toxicity using survival and growth tests for the sediment-dwelling chironomid *Chironomus riparius* and survival and reproduction tests for the daphniid *Daphnia magna*. This reconstruction of toxicity risk from multiple pollutants to sediment–dwelling biota (i.e. palaeotoxicity) should not be confused with paleoecotoxicology (sensu Herkovits 2001) which considers the impacts of chemical and physical stressors on the interpretation of the fossil record nor sensu Zeng (2014) which determines biological assemblage changes as a result of pesticide applications, metal(loid) contamination (Thienpoint et al. 2016) or other stressors (Korosi et al. 2017).

## Methods

### Study sites and sediment coring

**Fig. 1:**
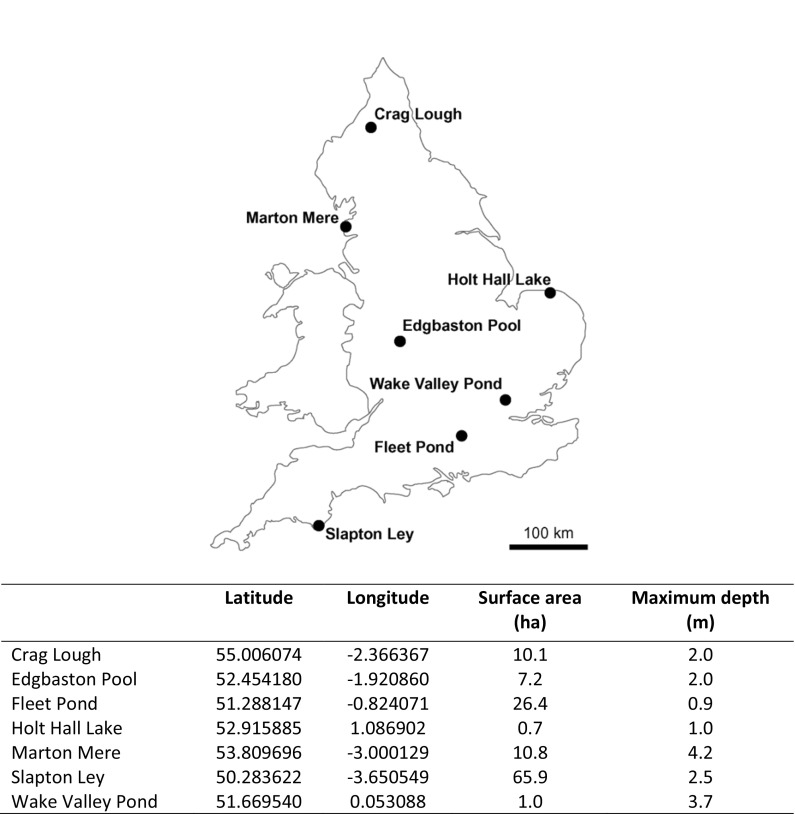
Study sites. Locational and morphological information

The 7 study lakes represent a diverse range of water bodies across England, UK, from urban ponds (Edgbaston Pool; Wake Valley Pond; Fleet Pond) and semi-urban sites draining urban areas (Holt Hall Lake; Marton Mere) to those in more rural settings (Crag Lough; Slapton Ley) (Fig. 1). These lakes have been subject to a variety of pressures, reflecting the range of multiple stressors that have determined the contemporary status of many lakes. Site descriptions are provided in Turner et al. (2013) and, briefly, also in Supplementary Information (S1). A sediment core was collected from the deepest part of each lake using a wide-diameter piston corer (Livingstone 1955) fitted with a polycarbonate tube with an internal diameter of 71 mm. Cores were sliced vertically in the field at 1-cm intervals. Samples were stored cool (4 °C) before being returned to the laboratory and freeze-dried prior to analysis.

### Radiometric dating

Sediment core samples were analysed for ^210^Pb, ^226^Ra, ^137^Cs and ^241^Am by direct gamma assay using ORTEC HPGe GWL series well-type coaxial low background intrinsic germanium detectors. Lead-210 was determined via its gamma emissions at 46.5 keV, and ^226^Ra by the 295 keV and 352 keV gamma rays emitted by its daughter isotope ^214^Pb following 3 weeks storage in sealed containers to allow radioactive equilibration for ^222^Rn and ^226^Ra. Caesium-137 and ^241^Am were measured by their emissions at 662 and 59.5 keV, respectively. The absolute efficiencies of the detector were determined using calibrated sources and sediment samples of known activity. Corrections were made for self-absorption of low energy gamma rays within the sample (Appleby et al. 1992). Unsupported ^210^Pb, from atmospheric deposition, was calculated by subtracting supported ^210^Pb (which derives from in situ decay of ^226^Ra) from total ^210^Pb. Final sediment chronologies were determined from ^210^Pb records using constant rate of supply (CRS) or constant initial concentration (CIC) models (Appleby 2001), in combination with ^137^Cs and ^241^Am profiles.

### Lithostratigraphic, trace metal and persistent organic pollutant analyses

Lithostratigraphic analyses (bulk wet density; water content; loss-on-ignition at 550 °C as an estimate of organic matter content) were undertaken on each sediment sample following standard methods (Dean 1974; Heiri et al. 2001). Geochemical and trace element analyses were undertaken using an X-ray fluorescence spectrophotometer (Spectro-X Lab 2000) while mercury (Hg) was analysed using cold vapour-atomic fluorescence spectrometry (CV-AFS) following an aqua regia digest. Details are provided in Supplementary Information (S2). Details of persistent organic pollutants (POPs) analyses are provided in Yang et al. (2016) and Supplementary Information (S2). Briefly, freeze-dried and homogenized sediment (typically 5 g, accurately weighed; pooled into 5-year increments based on radiometric dating) were treated with ^13^C-labelled internal standards and extracted using hexane and acetone (1:1, v/v). Extracts were purified prior to instrumental analysis. Tri-through-hexa-BDEs and PCBs were determined using an Agilent 6850-5975 GC-MSD, while BDE-183 and 209 analysis was conducted via LC-APPI-MS/MS. Determination of α-, β- and γ-HBCD used the same LC–MS/MS system used for BDEs − 183 and − 209. Although trace metal analysis was undertaken at a higher resolution than for POPs, trace metal data for palaeotoxicity calculations were amalgamated into the same 5-year periods as for POPs (c.1950–present) and on a decadal basis for pre-1950 time periods.

### Biological toxicity tests

Two sediment toxicity tests were completed: a 10-day chironomid survival and growth and a 7-day cladoceran survival and reproduction, according to ASTM (2000) and US EPA (Norberg King 1999; US EPA 2002). These were undertaken using surface sediments (5 cm) from each of the lakes collected at the same time as the sediment cores. Control and lake sediment samples for all screening toxicity tests were replicated five times with 10 organisms per replicate chamber. Both larval midges (*Chironomus riparius*) and juvenile cladocerans (*Daphnia magna*) were obtained from in-house cultures. Chironomids were first instar larvae ranging from 1 to 3 days post-hatch, while cladocerans (were 5 days old at study initiation. Statistical significance for animal survival was determined calculating differences from controls at the *p* = 0.05 level. Full details are provided in Supplementary Information (S2).

### Assessment of toxic risk

Most approaches to assessing mixtures of contaminants use a Relative Potency Factor (RPF) (Heys et al. 2016), whereby a measured concentration of contaminant is normalized to a standard concentration indicative of harm to an organism, followed by a method (usually a sum or a mean) to combine these individual factors into an overall metric. These RPF approaches for sediments include:

- Probable Effects Concentration Quotients (PEC-Qs) where sediment concentrations are normalized to the PEC (Long and Macdonald 1998) before taking a mean for groups of contaminants (e.g. metals; POPs) (Long et al. 2006);
- Toxic Units (TUs) where the measured concentration is normalized to a toxic endpoint identifier such as a 50% Effects Concentration (EC_50_) or a No Observed Effect Concentration (NOEC) and then summed to produce an overall ΣTU (Enserink et al. 1991; Lahr et al. 2003; Rasmussen et al. 2015; Heys et al. 2016; de Castro-Català et al. 2016);
- Mean Effects Range Median Quotients (M-ERM-Qs) where measured sediment metal concentrations are normalized to a defined ERM (Wang et al. 2015) and then averaged;
- Hazard Quotients (HQs) (Feng et al. 2011; Wang et al. 2015) which normalize measured concentrations by a guideline set at the ‘Effects Range Lower’ (ERL) (Long et al. 2000);
- Risk Quotients (RQs) which normalizes measured concentrations of biocides in sediments by the predicted no effect concentration (PNEC), although no combination of individual biocide quotients has been reported (e.g. Liu et al. 2015).

The RPF approach has also been employed with other ecological compartments. Kuzmanovic et al. (2016) used the toxic units approach to assess mixtures of metals, organic pollutants and pharmaceuticals using measured water concentrations rather than sediments, while Rasmussen et al. (2015) did the same for combinations of pesticides in both sediments as well as waters, normalizing the former to 96 h EC_50_ for acute mortality in sediment-dwelling chironomids and the latter to the 96 h EC_50_ for growth inhibition in green algae.

Alternative approaches to RPF are rare. Singh et al. (2002) (and later, also Diop et al. 2015) use a Sediment Pollution Index (SPI) for mixtures of metals which is defined as the linear sum of metal enrichment factors (EFs; measured sediment concentrations normalized to an average shale concentration) weighted by an assumed toxicity for that metal. So, SPI = Σ(EF_Me_ × *W*_Me_)/Σ*W*_Me_, where *W*_Me_ is the toxicity weighting of 1 for Cr and Zn, 2 for Ni and Cu, 5 for Pb and 300 for Cd. Tomlinson et al. (1980) also combine measured metals concentrations, although within biota, into a Pollution Load Index (PLI). This is a variation on the RPF approach as a contamination factor (CF) is produced for each metal, defined as the measured concentration in an organism normalized to a baseline reference concentration from a “clean” site. The CFs are then combined into a PLI by taking the nth root of the product of highest n CFs, i.e. PLI = [(CF_1_ × CF_2_ … CF_n_)]^root n.^

Finally, some studies combine metals in sediment pore waters into a Toxicity Index (TI) after normalizing extractable metal concentrations to acid volatile sulphide (AVS) (Roig et al. 2015; Diop et al. 2015). This approach to toxicity is assumed to measure bioavailable metal concentrations although whole sediment bioassays have shown more toxicity than pore water tests. Further, AVS can change with organic matter inputs and therefore could change down a sediment core through time as inputs change and decomposition occurs (Roig et al. 2015). While pore waters may represent a primary exposure route for nonpolar organic compounds and metals to sediment-dwelling organisms, some authors argue that they overestimate toxicity of more soluble compounds in sediments (e.g. metals) compared to less soluble organic compounds (Lahr et al. 2003).

In this study, we use the mean Probable Effects Concentration Quotients (mean PEC-Q) approach as it provides an effective means by which to combine a broad range of trace metals and organic pollutants. PEC-Qs for individual trace metals and ΣPCBs were calculated by dividing the measured sample concentration by the respective consensus-PEC where possible (MacDonald et al. 2000; Ingersoll et al. 2001). For PBDEs and HBCD, no consensus-PECs were available, so Canadian Federal Environmental Quality Guidelines (FEQGs) for Penta-BDE and BDE-209 of 0.4 and 19 ng g^−1^ were used, respectively (Environment Canada 2013). An equivalent guideline for ΣPBDEs was not available. However, Penta-BDE components (chiefly BDE-47 and 99) are considered more toxic than higher brominated congeners and therefore are of greater concern (see, for example, US EPA Integrated Risk Information System; US EPA 2008a, b, c) so reference to these rather than ΣPBDEs may represent a better assessment of biological risk from these compounds. Similarly, no consensus-PEC was available for HBCD so the Canadian FEQG (1600 ng g^−1^) was used (Environment and Climate Change Canada 2016).

Following Ingersoll et al. (2001) we calculated a ‘PEC-Q Mean-Metals’; a ‘PEC-Q Mean-POPs’ and a ‘PEC-Q Mean-All’, the latter giving an equal weighting to the contributions of these diverse pollutant groups assuming they exert toxic effects through independent action, a more likely ‘real-world situation’ (Norwood et al. 2003; Walter et al. 2002). A mean PEC quotient > 0.5 has been found to coincide with a consistent increase in freshwater sediment toxicity (Ingersoll et al. 2001) while Rippey et al. (2008) found biological effects (for PEC-Q-Mean-Metals) when the quotient > 2.0. Here, we use these two thresholds to suggest ‘possible’ and ‘probable’ detrimental biological effects, respectively. A critical review of the use of mean Sediment Quality Guideline Quotients is given in Long et al. (2006). In short, the advantage of using means of quotients (over sums) is that they are subject to less variability if different numbers of chemicals are measured in different samples, for example where multiple gradients of multiple chemicals exist in a study area (or through time down a sediment core). However, there is an assumption that the relative risk from samples with different mixtures of chemicals but the same mean-quotient would be the same.

## Results and discussion

### Sediment chronology and diagenetic considerations

Sediment core depth/age profiles and sediment accumulation rate data for each lake are presented in Supplementary Information (S3). Radiometric chronologies extend back to the nineteenth century for all cores except for Edgbaston Pool where the earliest date was 1944 (± 16 years) at 36 cm depth. Sediment accumulation rates in these lakes generally increase through time, in agreement with many European lake types (Rose et al. 2011), except for Edgbaston Pool and Crag Lough; the latter exhibiting a major peak in 1950 (± 3 years) possibly due to a rock-fall from the cliffs above redistributing contemporary sediments (Turner et al. 2013).

Sediment cores have an advantage over direct monitoring as it is possible to obtain long temporal records of contamination without the need for regular measurement over decadal timescales (Battarbee et al. 2014). However, there is a need to consider how reliable this historical record is, both to ensure that the sediments are not disturbed but also that pollutant concentrations ascribed to a dated sediment sample are equivalent to concentrations when that sample was at, or near, the sediment surface. In many sediment studies, such issues are not critical as directions and rates of change are more important, but here, in evaluating likelihood of toxic effects to aquatic biota, post-burial diagenetic effects are an important consideration.

The well-resolved sediment profiles for individual contaminants (Supplementary Information S4 and S5) and background geochemical data (Supplementary Information S3) show no indication of large-scale disturbance apart from the 1950 sediment event at Crag Lough. Furthermore, most of the cores have well-resolved 1963 ^137^Cs peaks derived from the maximum fallout from nuclear weapons testing (Supplementary Information S3) indicating that the sediments have been relatively unmixed since at least the 1960s. The 1963 ^137^Cs peaks in the cores from Holt Hall and Slapton Ley are less well-resolved, but still distinct, suggesting that these sediments may have been subject to only limited post-depositional mixing. In terms of diagenetic processes affecting trace metal movement within sediment cores, rapid exchange of some metals may occur between surface organic sediment and the water column due to the decay of freshly deposited organic debris but appears to have little significance for the longer term sediment record (Boyle 2001a). Furthermore, of the elements investigated here, only Cr may be susceptible to changes in solubility over the range of redox conditions normally found in lake sediments (Boyle 2001a). For other metals, redox effects are indirect and any migration within the sediment column results from the presence of Mn and Fe oxyhydroxide peaks in lakes with exceptionally low sediment accumulation rates (10 mg cm^−2^ year^−1^) (Boyle 2001b). Sediment accumulation rates in all cores in this study are far higher than this (Supplementary Information S3), and hence it is very unlikely that redox effects have had a major influence.

With respect to POPs, while some post-depositional alteration of sediment records via processes such as within-core diffusion and degradation is known or suspected (Sanders et al. 1992), the validity of studying dated lake sediment cores for historical monitoring of organochlorine POPs has been demonstrated (Eisenreich et al. 1989). Our previous report of trends in these English lakes reveals temporal variation in deposition of PBDEs, PCBs, and HBCDs that closely reflect trends in the manufacture and use of these compounds in the UK (Yang et al. 2016). In all instances, concentrations of target POPs in core slices dated to decades prior to c.1950 were below detection limits. The only exception was for PCBs, which were found in a core slice from Edgbaston Pool dated to the early-1900s, at a concentration that was between 3.1 and 17% of that determined in later sediment samples. Overall, our study suggests that post-depositional modification processes exert only a minor influence on sediment records of POPs.

### Sediment quality guideline exceedances: individual pollutants

Trace metal and POPs concentrations for each lake core are presented with TEC and PEC values in Supplementary Information S4 and S5, respectively. Threshold Effects Concentrations for all trace metals are exceeded at all sites (except Cu at Crag Lough where a single exceedance occurs at c. 100 cm). For the rural sites (Crag Lough and Slapton Ley), PEC exceedance is limited to Ni and Cr and only at lower depths. By contrast, the urban lakes exceed PEC values for all trace metals across a considerable range of depths, and especially in upper sediments, except for Cr (Edgbaston Pool; Wake Valley Pond), Hg (Fleet Pond; Wake Valley Pond) and Cu (Fleet Pond; Wake Valley Pond). For the other lakes, PECs are exceeded for all trace metals at Marton Mere while at Holt Hall Lake only the PEC for Pb is exceeded to any extent, as there is only a single exceedance for Ni in deeper sediments. For POPs, the TEC for ΣPCBs is only exceeded at Edgbaston Pool and Marton Mere. No concentrations approach the PEC for ΣPCBs at any site. Similarly, the FEQG for HBCD is not exceeded at any site. However, the FEQG of ΣPenta-BDEs is exceeded at all sites except Slapton Ley while the FEQG for BDE-209 is exceeded in Edgbaston Pool, Marton Mere and the surface sediment of Fleet Pond. However, BDE-209 concentrations are increasing at all sites and further exceedances may occur if these trends continue.

Some trace metal exceedances in upper sediments are considerable, with Pb concentrations exceeding its PEC by more than a factor of 10 in Edgbaston Pool, > 4 in Holt Hall Lake and > 3 in Wake Valley Pond; Zn concentrations exceed PEC by factors of 6.5 and 3.8 in Edgbaston Pool and Wake Valley Pond, respectively; Cu exceeds its PEC by a factor of 3 in Edgbaston Pool and Ni by > 5 in Wake Valley Pond. These data indicate that the probability of detrimental effects to sediment-dwelling organisms is both considerable and extensive, especially in urban lakes, due to the sub-surface concentrations of contaminants, particularly trace metals. While these data provide an indication of effects due to individual contaminants, there is a need to consider their effects in combination as a more realistic assessment of exposure.

### Reconstructing palaeotoxicity for multiple pollutants

**Fig. 2:**
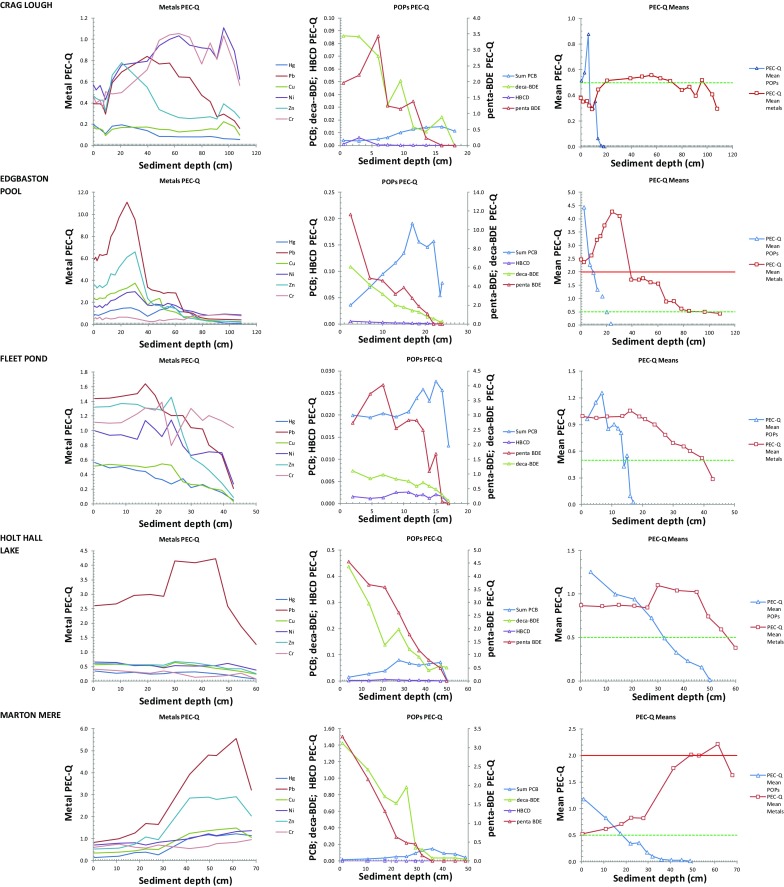

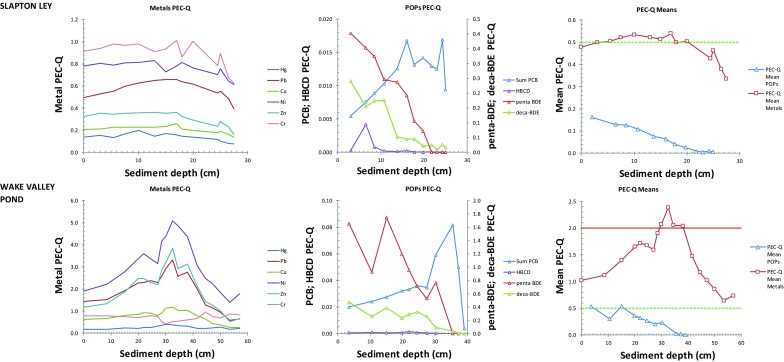
Probable Effects Concentration Quotients (PEC-Qs) plotted against depth for individual trace metals (left); POPs (centre) and Mean-metals and Mean-POPs (right). Horizontal green and red lines are PEC-Qs of 0.5 and 2.0, respectively, representing ‘possible’ and ‘probable’ detrimental biological effects, respectively

Probable Effects Concentration quotients (PEC-Qs) for individual trace metals and POPs for each lake are presented in Fig. 2, together with the PEC-Q Mean-metals and PEC-Q Mean-POPs. Following Ingersoll et al. (2001), mean quotients may be used to classify samples as toxic or non-toxic, while quotients for individual substances can be used to identify those metals and/or POPs contributing to that toxicity. A consistent increase in toxicity is observed where mean PEC-Q exceeds 0.5 whichever form of the quotient is used (Ingersoll et al. 2001; Rippey et al. 2008) while a PEC-Q Mean-Metals value > 2 in freshwater sediment leads to detrimental biological effects such as a decrease in the number of taxa (Dave 1992). ‘Total’ concentrations of contaminants are used rather than any estimate of the bioavailable fraction as these are the values employed in SQGs as well as consensus-PEC determinations and hence PEC-Q calculations. The PEC approach therefore reconstructs toxic risk (i.e. palaeotoxicity) rather than bioavailability (Besser et al. 2015).

PEC-Q Mean-Metals exceeds 0.5 in all lake cores. At the rural sites (Crag Lough; Slapton Ley), this exceedance is marginal and appears to be due to contributions from Cr, Ni and possibly Pb (Fig. 2). While the 0.5 threshold is exceeded at both sites, it has been declining over the uppermost 20 cm and PEC-Q Mean-Metals values fall below 0.5 in surface sediments. PEC-Q Mean-POPs at these sites increases in the uppermost 20 cm, dramatically so in Crag Lough, such that PEC-Q Mean-POPs exceeds PEC-Q Mean-Metals at about 7 cm depth, indicating a greater impact from POPs (principally due to Penta- and Deca-BDE) at the site for the first time. By contrast, two of the urban lakes (Wake Valley Pond; Edgbaston Pool) exceed the upper effects boundary (> 2) for PEC-Q Mean-Metals. At Edgbaston Pool, this exceedance is by more than a factor of 2 and appears mainly due to high Pb and Zn concentrations. Values decline in the upper 20 cm but although they remain > 2, PEC-Q Mean-Metals is exceeded by PEC-Q Mean-POPs in the upper 10 cm, again due to Penta- and Deca-BDE (Fig. 2). At Wake Valley Pond, the high PEC-Q Mean-Metals due to Ni, Zn and Pb has also declined but remains at around 1.0 in surface sediments. Here, although PEC-Q Mean-POPs has increased steadily in the upper 20 cm, it has only recently reached 0.5. At Marton Mere, PEC-Q Mean-Metals also reaches 2, mainly due to Pb and Zn but only at lower sediment depths. Mean-Metals values decline in upper sediments and are exceeded by PEC-Q Mean-POPs at around 15 cm. Again, the upward trajectory of PEC-Q Mean-POPs exceeds 0.5 above 20 cm, mainly as a result of increasing PBDEs. At Fleet Pond and Holt Hall Lake, PEC-Q Mean-Metals exceeds 0.5 through most of the core. At Fleet Pond, a range of metals contribute (Pb, Zn, Ni, Cr) while at Holt Hall Lake, exceedance is almost entirely driven by high Pb. At both sites, PEC-Q Mean-POPs exceeds PEC-Q Mean-Metals in upper sediments, though while this is due to PBDEs at Holt Hall Lake (similar to other lakes), at Fleet Pond, BDE-209 is less important and ΣPCBs also contributes.

In summary, PEC-Q Mean values of 0.5 are exceeded at all sites with values of 2.0 observed at all urban lakes. Hence, greater risk from toxic effects may be expected at these. At some sites (e.g. Edgbaston Pool) mean values for both metals and POPs are > 4 mainly due to high concentrations of Pb, and both Penta- and Deca-BDE. The same pollutants appear to be responsible for most of the predicted toxicity at other sites, both urban and, to a lesser extent, rural. At all sites, PEC-Q Mean-Metals has declined in recent sediments. By contrast, PEC-Q Mean-POPs are increasing and exceed 0.5 at all sites except Slapton Ley. At 5 of 7 sites, PEC-Q Mean-POPs has overtaken PEC-Q Mean-Metals in recent sediments as the main agent of toxicity risk.

### Historical trends of mean PEC-Qs

**Fig. 3:**
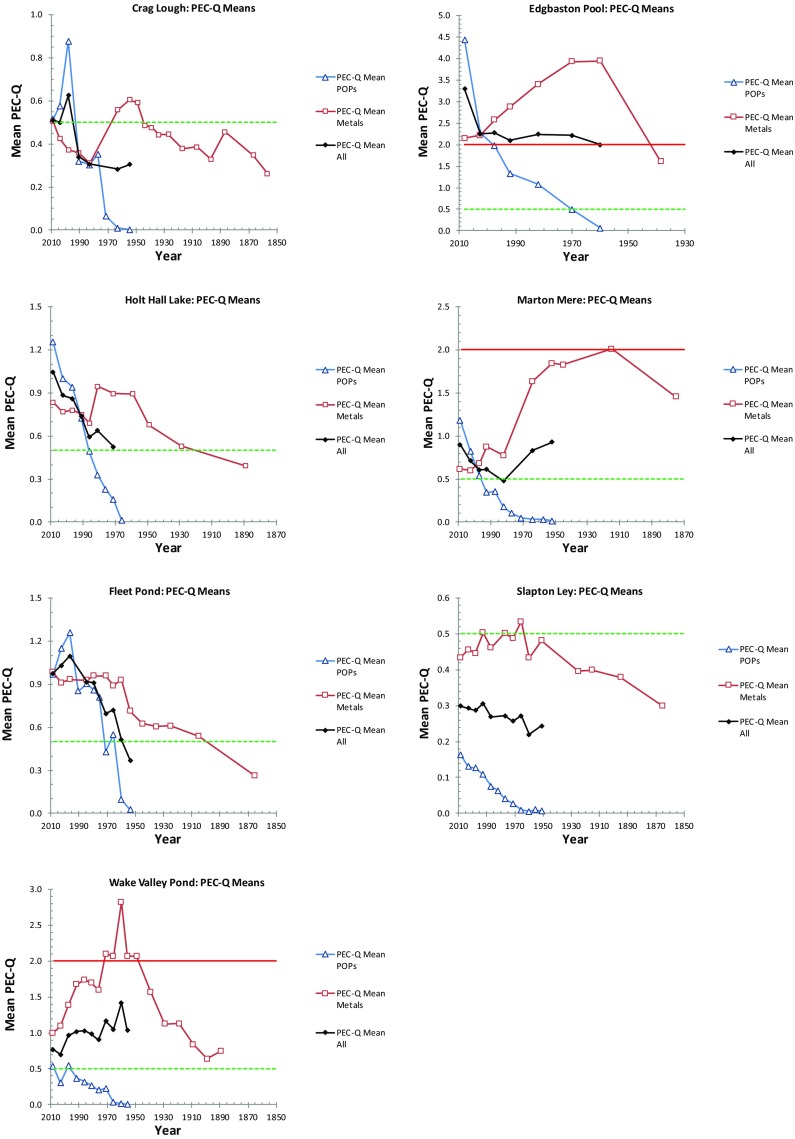
Mean PEC-Qs (Metals; POPs; All) for all lakes plotted against date. Horizontal green and red lines are PEC-Qs of 0.5 and 2.0, respectively, representing ‘possible’ and ‘probable’ detrimental biological effects, respectively

Plotting the PEC-Q data on a chronological axis (Fig. 3) allows a reconstruction of temporal trends in toxicity over the last 150 years. At rural sites, PEC-Q Mean-Metals only exceeds 0.5 very briefly in the 1960s at Slapton Ley and for a period of about 20 years from c.1945 at Crag Lough. At both sites, values then decline through to more recent times although at Crag Lough values increase once again from 1990 possibly as a result of recent remobilisation of a range of metals from catchment soils (Rose et al. 2012). At Edgbaston Pool, Marton Mere and Wake Valley Pond, PEC-Q Mean-Metals exceeds 0.5 throughout the dateable period of the core (from the late-nineteenth century at Marton Mere and Wake Valley Pond; from the 1940s at Edgbaston Pool), while at Fleet Pond and Holt Hall this exceedance starts from the early decades of the twentieth century and continues to the present. PEC-Q Mean-Metals peak in the 1960s–1970s at all sites except at Marton Mere where the peak is earlier (1910s) and at Fleet Pond where these higher values remain to the present. At Marton Mere, this earlier contamination is likely due to the site being exposed to local urban waste as it was formerly connected to the drainage system for Blackpool (Turner et al. 2013) although later this drainage bypassed the site following improvement measures. At Edgbaston Pool, Wake Valley Pond and Marton Mere, the upper PEC-Q threshold (2.0) is also exceeded, at Edgbaston for over 60 years (1940–present; Fig. 3) although concentrations are currently decreasing. While these declines have been considerable and rapid (0.15–0.45 PEC-Q units per decade), Marton Mere and Wake Valley Pond still exceed the 0.5 threshold and Edgbaston Pool still remains above 2.0 indicating continuing, and significant toxicity risk from trace metals.

At all sites, PEC-Q Mean-POPs values have increased rapidly since 1950s and (apart from Slapton Ley) first exceed 0.5 between 1970s (Edgbaston Pool; Fleet Pond) and 1990s (Crag Lough; Marton Mere; Wake Valley Pond). Only at Edgbaston Pool has PEC-Q Mean-POPs exceeded 2.0. This first occurred in the 1990s and has continued to increase to > 4.0 in surface sediments. If current trends continue, the upper (2.0) threshold will also be exceeded at Holt Hall Lake and Marton Mere within 20 years. With the decline in metals concentrations and the rapid increase in, especially, PBDEs, PEC-Q Mean-POPs first exceeds that of metals between the 1990s and 2005 at all sites except Wake Valley Pond and Slapton Ley. However, if current temporal trends continue, this would also happen in around 10 and 50 years at Wake Valley Pond and Slapton Ley, respectively. The rapidly increasing trajectories for the PEC-Q Mean-POPs therefore now dominate the records in these lakes and drive the overall PEC-Q Mean-All. Consequently, PEC-Q Mean-All is currently increasing rapidly at 4 out of 7 lakes and only appears to decline at Wake Valley Pond, driven by a dramatic decline in metals inputs (Ni, Zn, Pb). Elsewhere, at Fleet Pond and Crag Lough, PEC-Q Mean-All has declined in most recent sediments but whether this is temporary or a more long-term pattern is currently uncertain.

At Edgbaston Pool, PEC-Q Mean values have exceeded the upper 2.0 threshold continuously since the mid-1940s, initially as a result of trace metals (principally Pb and Zn) and more recently due to PBDEs counteracting the decline in metals inputs. This is the only site for which PEC-Q Mean-All exceeds this upper threshold and hence might be expected to be the one at which detrimental biological impacts are most likely to have occurred. However, long exposure to high levels of contaminants could also result in the development of tolerance. In south-west England, mine-water outwash has resulted in elevated trace metal concentrations in rivers since the Medieval period resulting in genetically distinct Brown trout (*Salmo trutta* L.) populations separated by a chemical barrier of metal contamination (Paris et al. 2015). Metal concentrations in some parts of the river are now toxic to ‘metal-naïve’ fish from other parts of the same river. It maybe that in lakes continuously contaminated over extensive periods, tolerance may have developed within sediment-dwelling organisms, especially those with short generation intervals, such as chironomids. However, such changes are also reversible and a study on the aquatic oligochaete *Limnodrilus hoffmeisteri*, Levinton et al. (2003) suggests that genetic resistance to metals may also be lost by sediment-dwelling biota following remediation, i.e. when the force for selection is removed.

### Comparisons between predicted risk and biological toxicity tests

Although PEC-Q Mean-All calculated toxicity risks are highest for the surface sediments from Edgbaston Pool this is not reflected in the biological toxicity tests (Table 1). For the chironomid tests, only surface sediments from Crag Lough showed a significant reduction in survival while all lakes except Fleet Pond and Wake Valley Pond showed a significant reduction in surviving chironomid growth compared to the control. For daphniids, results were similar. Again, only Crag Lough surface sediments showed a significant reduction in *Daphnia* survival compared to the control while all sites showed a statistically significant reduction in the number of young per adult.

**Table 1:** Results of toxicity tests for lake surface sediments

	*Chironomus riparius*		*Daphnia magna*		Predicted toxicity
	% survival	Growth	% survival	Reproduction	PEC-Q Mean-All
Control	80	1.12	100	19.5	–
Crag Lough	30*	0.70^†^	40*	6.1^†^	0.51
Edgbaston pool	91	0.70^†^	90	11.0^†^	3.29
Fleet pond	90	1.01	100	14.4^†^	0.97
Holt Hall lake	65	0.50^†^	80	8.6^†^	1.04
Marton Mere	96	0.89^†^	90	11.3^†^	0.90
Slapton Ley	86	0.62^†^	100	10.8^†^	0.30
Wake Valley Pond	84	1.22	90	8.8^†^	0.77

Percent survival and growth (as ash-free dry weight per organism in mg) of *Chironomus riparius* and percent survival and reproduction (as mean total young production per adult) for *Daphnia magna*

*Indicates a statistically significant reduction in % survival as compared to the control mean using Dunnett’s and Fisher’s exact test (*p* < 0.05)

^†^Indicates a statistically significant reduction in growth and reproduction tests using Dunnett’s test (*p* < 0.05). Also shown are PEC-Q Mean-All values for surface sediments. These are shaded by their exceedance of various thresholds: < 0.5 (green), 0.5–2.0 (yellow), > 2.0 (red)

The predicted toxicity of sediments to organisms may be influenced by a large number of factors including bioavailability (Long and MacDonald 1998), the presence of un-measured substances (Lahr et al. 2003; Long et al. 2006; Kuzmanovic et al. 2016) and other confounding variables including physicochemical factors which increase environmental stress on an organism (Korosi et al. 2017). These latter stressors, including pH, water chemistry, temperature and oxygen levels, can increase the sensitivity of individuals to toxicants by several orders of magnitude (Liess et al. 2016) such that biological effects may be amplified when an individual is weakened by unfavourable conditions. Calculations of PEC-Qs can also be hampered by substances for which no sediment quality guidelines are available (Long and MacDonald 1998). The toxic effects on biota by the sediments from Crag Lough, with respect to the other lakes, cannot be due to physicochemical confounding factors such as water chemistry, oxygen or food availability as these were kept the same for all tests. Therefore, it may be that the toxicity of Crag Lough sediments is due to the presence of a contaminant that was not included in our analyses. There are a large number of possibilities, but two groups not included were the polycyclic aromatic hydrocarbons (PAHs), produced from the combustion of organic matter, and organochlorine pesticides. While the use of organochlorines have been restricted in the UK for decades (HSE 2017) and banned since the Stockholm Convention in 2004, so also have PCBs and these were detected in the sediments of all sites (Supplementary Information S5). However, although both of these un-measured contaminant groups have been recorded at high concentrations in lake sediments (Sanders et al. 1992; Rippey et al. 2008), it is unclear why they would be in higher concentrations in Crag Lough, a remote lake surrounded by an agricultural catchment, compared to other lakes in urban settings. The remobilization of previously deposited contaminants from storage in catchment soils to both lakes and rivers is now widely recognized across upland UK (Rose et al. 2012; Rothwell et al. 2005, 2007). Of all the lakes in this study, the catchment of Crag Lough is most susceptible to catchment soil erosion as it is large, unafforested and grazed by cattle and sheep. It could be that enhanced soil erosion at the site, has resulted in increased inputs of un-measured contaminants from the catchment to the sediments thereby increasing toxic effects.

Finally, the presence of a contaminant in a mixture and its known toxicity is no guarantee of the effects when combined with other factors (Heys et al. 2016), and unless analysis is exhaustive, it is not possible to know which combinations of chemicals will arise in the environment (Malaj et al. 2014). Hence, it is possible that in contrast to the presence of an un-measured contaminant increasing the toxicity of the surface sediments in Crag Lough, some sedimentary parameter is increasing the bioavailability of contaminants in the sediments at this site but not at the others. Alternatively, it may be that other sediment characteristics are reducing the bioavailability of contaminants at Edgbaston Pool thereby lowering the toxic effects from the higher concentrations of contaminants in the sediments.

While the sensitivity of organisms to contaminants is affected by the presence of other stressors (Liess et al. 2016), aquatic biota, including those in sediments are known to have developed a genetic tolerance if exposure continues for many generations (Levinton et al. 2003; Paris et al. 2015). While such a tolerance may be expected to have developed at lakes with long histories of high levels of contamination such as Edgbaston Pool and Marton Mere, this cannot be the explanation for our observed results for Crag Lough as chironomids and daphniids used in the toxicity tests were laboratory-bred and therefore had identical tolerances. Conversely, however, it could be argued that there is some agreement between these tests. The PEC-Q Mean-All shows an elevated prediction of risk (> 0.5) at all sites except Slapton Ley while the biological toxicity tests show a reduction in chironomid growth for all sites except Wake Valley Pond and Fleet Pond and a reduction in *Daphnia* reproduction at all sites. Therefore, it maybe that a toxic effect is being both predicted and observed at these sites although there is insufficient discrimination along the predicted contamination gradient. The biological toxicity tests were standard 8–10-day assessments (Norberg King 1999; US EPA 2002), and it maybe that these exposures were not sufficiently long to differentiate between the contaminant levels at the study sites.

At present it is not possible to determine whether any discrepancy between the predicted toxicity risk and observed biological effects are due to the toxicity calculations (e.g. incomplete contamination assessment) or due to the biological tests (e.g. other sedimentary factors enhancing (Crag Lough) or suppressing (Edgbaston Pool) bioavailability or toxic effects). Further work is therefore required to explore these. This could include a more complete analysis of sediment contaminants and other sedimentary parameters; undertaking biological toxicity tests on sediments taken from throughout a sediment record; or analysis of lakes from across a greater contamination gradient.

## Conclusions

Exceedance of sediment quality guidelines for both trace metals and POPs was common especially in urban lakes where a range of trace metals and POPs were found to exceed PECs, often by factors of 2–5, suggesting enhanced risk to sediment-dwelling biota from these contaminants in isolation. At rural sites only the lower TEC thresholds were exceeded. Although the reconstructed palaeotoxicity data show marked reductions in risks to sediment-dwelling biota from trace metals in recent decades, remobilisation of previously deposited contaminants, stored in catchment soils (Rose et al. 2012; Yang and Smyntek 2014) could allow these to increase once again. In some cases the combined probability (PEC-Q Mean-All) remains above biological risk thresholds and the risk posed by rapidly increasing concentrations of POPs now exceeds that of trace metals at many sites. The rate and timing of this crossover in priorities will be important for contaminant management and mitigation at the site-specific level. In surface sediments, predicted toxicity assessments showed limited agreement with observed biological toxicity tests. This could be due to the presence of un-measured contaminants in the surface sediments and/or other confounding factors enhancing or mitigating for toxic effects at particular sites.

While in the current dataset, Pb and PBDEs appear to be the contaminants of most concern, in the past and present, respectively, the study did not include all contaminants (especially POPs) that might be expected to have a detrimental biological effect. Polycyclic aromatic hydrocarbons (PAHs) (Galloway et al. 2004), dioxins and furans (PCDD/Fs) (Cook et al. 2003) and other organochlorines (Sundberg et al. 2007), are just some of the POPs that could also be considered. It is unlikely that any study could analyse a fully exhaustive list of potential contaminants and hence calculations of exposure are always likely be underestimates (Long and McDonald 1998; Kuzmanovic et al. 2016). However, adopting the palaeotoxicity approach and recognizing the rate and scale of changing risk to sediment-dwelling organisms, determined from deposited sediments, would enhance the level of protection currently afforded these organisms as well as higher-level aquatic species and those dependent upon them.

## Electronic supplementary material

Below is the link to the electronic supplementary material.

Supplementary material 1 (DOCX 776 kb)
