# Supplementary material for: Palaeotoxicity: reconstructing the risk of multiple sedimentary pollutants to freshwater organisms
Source: Environ Geochem Health. 2018 Mar 2;40(4):1667–82. doi: 10.1007/s10653-018-0080-5 (PMC6061110; doi:10.1007/s10653-018-0080-5)
Supplement: Supplementary file 1 — Supplementary material 1 (DOCX 776 kb) [file 10653_2018_80_MOESM1_ESM.docx]

**Palaeotoxicity: reconstructing the risk of multiple sedimentary pollutants to freshwater organisms**

**Supplementary Information**

Neil L. Rose^†,*^, Simon D. Turner^†^, Handong Yang^†^, Congqiao Yang^§,‡^, Charlotte Hall^†^ and Stuart Harrad ^‡^

^†^ Environmental Change Research Centre, Department of Geography, University College London, Gower St, London WC1E 6BT, UK

^§^ Current address: Department of Earth Sciences, University of Toronto, Toronto, Ontario M5S 3B1, Canada

^‡^ School of Geography, Earth & Environmental Sciences, University of Birmingham, Birmingham B15 2TT, UK

^*^ author for correspondence

**Includes:**

S1 Site descriptions Page S2

S2 Detailed analytical methods Page S4

S3 Core chronological and background geochemical data Page S9

S4 Trace metal data Page S11

S5 Persistent organic pollutants data Page S13

References Page S14

**Supplementary Information S1: Site descriptions**

**Crag Lough (Northumberland)** is a lake at the foot of High Shield Crag, near Housesteads. The rock cliffs forming the southern edge of the lake are an exposure of a 296 million year old volcanic intrusion, the Whin Sill. Running along the top of the cliffs and overlooking the lake is the course of the Roman constructed Hadrian’s Wall popular with walkers. The lake and its wetland margins are a Site of Special Scientific Interest (SSSI) managed by the National Trust, and sustaining a nationally important diversity of plant species. The site is surrounded by farmland, primarily for the grazing of sheep and cattle.

**Edgbaston Pool (Birmingham)** is only 3 km from the centre of Birmingham. The lake was formed by damming a small stream (Chad Brook) that enters from the north. This occurred as early as the 16th century to provide water power for various industrial mills. The lake was once surrounded by industry, but is now flanked by Winterborne Botanic Gardens to the west and the Edgbaston Golf Course to the east. It was given SSSI status in 1986 and has diverse woodland and wetland habitats around its margins.

**Holt Hall Lake (Holt, Norfolk)** probably owes its origin to fish ponds constructed in the Middle Ages. From the mid-19th century, the lake was managed with the gardens as an ornamental water body. Prior to the early 1960s, much of the effluent and waste water from the local area entered the lake but some primary treatment took place from the 1960s to the 1990s with full connection to the mains sewerage in 2007. The lake is very shallow and in-filling rapidly. Considerable levels of leaf-litter are deposited every year from the woodlands along the eastern bank and filamentous algae cover the water surface each summer.

**Marton Mere (Blackpool)** formed at the end of the last Ice Age as frozen ground collapsed and a large, deep depression filled with water (Turner et al 2013). The Mere was gradually drained throughout the 18^th^ century, and more extensively by around 1850 when a main dyke was cut (Clarke, 1969). It was also used as a refuse tip until 1972 but designated a SSSI in 1974 and became a Local Nature Reserve in 1991. It is an important site for native and migratory birds. There is a large caravan site on the southern shore, but access to the lake itself is restricted.

**Fleet Pond (Fleet, Hampshire)** was originally a medieval fishpond, made by damming streams in a wide valley. The historical extent of the water body has been affected by the changing nature of local land-use. A railway was constructed in the 1830s passing through the catchment, and in the early 20^th^ century it was used as a testing ground for seaplanes. The pond was completely drained in the 1940s to avoid its refection in moonlight guiding enemy aircraft during World War 2. Continued inputs of contaminated water from commercial and domestic sources and soil erosion in the catchment, especially in recent decades, have reduced water depth, leading to increased sediment resuspension and reduced water clarity. A restoration programme has recently been undertaken.

**Slapton Ley (Devon)** is the largest natural freshwater lake in southwest England. It was formed by rising sea levels and coastal sediment damming a small estuary by a shingle barrier around 3000 years ago. The large (219 ha) freshwater lake and wetland area behind the shingle barrier creates a diverse and nationally important range of habitats, which forms the Slapton Ley National Nature Reserve. The Field Studies Council has used the lake and Reserve as an outdoor laboratory for over 50 years, and thousands of people visit every year. The catchment is primarily agricultural land devoted to the grazing of cattle and sheep.

**Wake Valley Pond (North London)** is a small (approx. 1 ha) pond in Epping Forest. The current pond was created in the 19th century by the construction of a road embankment (the current A104) across the valley. It is a well-known lake for dragonflies and amphibians. The lake is a SSSI, visited extensively by anglers and visitors to Epping Forest. The catchment is entirely deciduous woodland.

**Supplementary Information S2: Detailed analytical methods.**

Geochemical elements and trace metals

Weighed (4 d.p.) freeze-dried milled sediment (~2 g) was measured using an X-ray fluorescence spectrophotometer (Spectro-X Lab 2000) for trace metals (Pb, Cu, Ni, Zn, Cr) and other geochemical elements. A certified reference sediment sample of similar mass was included in each analytical run (Buffalo River Sediment NIST 8704 for all metals except Cu; NIST 2704 for Cu - Epstein et al. 1989) with mean recovery rates of 95.2% (Cr) - 109.8% (Cu) (N = 19). For mercury (Hg) analysis, 0.2 g of freeze-dried sediment was weighed into a 50 mL polypropylene DigiTUBE (SCP Science). 8 mL aqua regia were added to each and gradually heated on a hotplate to 100 °C to avoid violent reaction. The sample was then digested for another 1.5 h and allowed to cool. The digested solution was diluted to 50 mL using distilled deionised water. Standard reference stream sediment (GBW07305; certified Hg value 100 ± 10 ng g^-1^; our measured mean value 100.3 ng g^-1^; RSD=4.5 ng g^-1^; N=15) and sample blanks were digested with every 20 samples. Mercury concentrations were measured by cold vapour-atomic fluorescence spectrometry (CV-AFS) following reduction with SnCl_2_. Standard solutions and quality control blanks were measured after every five samples to monitor measurement stability.

Persistent organic pollutants

Freeze-dried and homogenised sediment (typically 5 g, accurately weighed; pooled into 5-year increments based on radiometric dating) were treated with ^13^C-labelled BDE-28, BDE-47, BDE-99, BDE-153, and BDE-209 (Wellington Laboratories, Canada), PCBs 34, 62, 119, 131, and 173 (Dr. Ehrenstorfer GmbH, Germany), and ^13^C_12_-labelled α-, β-, and γ-HBCDs (Wellington Laboratories, Canada) as internal (or surrogate) standards. Samples were extracted using a Speed Extractor (E-916 mode, BÜCHI, Switzerland) with hexane and acetone (1:1, v/v) at 100 ˚C under 120 bar for 3 cycles x 5 min per cycle. Extracts were collected in 240 mL glass collection vials containing 5 g pre-cleaned copper wire (for sulphur removal) and stored overnight. Concentrated crude extracts (1 mL) were purified prior to instrumental analysis by passing through a hydrophilic PTFE filter before purification via gel permeation chromatography, followed by florisil chromatography and concentration. Tri-through-hexa-BDEs and PCBs were determined using an Agilent 6850-5975 GC-MSD operated in electron ionisation, selective ion monitoring (EI^+^-SIM) mode fitted with a VF-5 ms capillary column (30 m x 0.25 mm x 0.25 *µ*m) (Harrad and Hunter 2006). Determination of BDE-183 and -209 was conducted via LC-APPI-MS/MS, using a dual pump Shimadzu LC-20AB Prominence liquid chromatograph interfaced with a Sciex API 2000 triple quadrupole mass spectrometer (Abdallah et al. 2009). Determination of HBCDs followed Harrad et al. (2009) with minor alterations in mobile phase composition. Target compounds (α-, β- and γ-HBCD) were determined using the same LC-MS/MS system used for BDEs -183 and -209, operated in electrospray negative ionisation mode (ESI^-^). Chromatographic resolution of α-, β- and γ-HBCDs was achieved on a Varian Pursuit XRS3 C18 reversed phase analytical column (150 mm × 2 mm i.d., 3 μm particle size). Method accuracy was assessed via triplicate analysis of NIST SRM2585 (Organics in House Dust). Results showed very good agreement with certified (PCBs and PBDEs) and indicative (HBCDs) values. Recoveries of the ^13^C_12_-labelled BDE standards added to the samples prior to extraction ranged from 91.5 to 107 % with an average value of 97.4 %. One method blank was analysed alongside each batch of 11 samples. No target PCBs, PBDEs, and HBCDs were found at detectable levels in any blank. Based on a typical sample mass of 5 g, method detection limits were: 0.001, 0.001, 0.002 ng g^-1^ dw for α-, β- and γ-HBCD, respectively; 0.003-0.05 ng g^-1^ dw for PBDEs; and typically 0.03 ng g^-1^ dw for PCBs.

Biological toxicity tests

Two sediment toxicity tests were completed; 10-day chironomid survival and growth and 7-day cladoceran survival and reproduction, according to ASTM (2000) and US EPA (Norberg King 2000; US EPA 2002). These were undertaken using surface sediments (5cm) from each of the lakes collected at the same time as the sediment cores. For both tests, overlying water was prepared by blending well water with well water treated by reverse-osmosis to produce water with a hardness of 130 to 160 mg CaCO_3_ L^-1^. Control sediment was formulated by mixing the following ratio of constituents: 75 % fine industrial sand, 20 % kaolin clay, and 5 % sphagnum peat. The sediment constituents were mixed based on dry weight equivalents. The peat moss was sieved to a finely ground consistency and did not contain any visible plant remains. Calcium carbonate was added to the artificial sediment to adjust the pH to 7.0 ± 0.5 units. The mixed sediment was stored dry until it was hydrated to approximately 39 % of its dry weight just prior to its use. Control and lake sediment samples for all screening toxicity tests were replicated five times with 10 organisms per replicate chamber. Test chambers were maintained in a temperature-controlled water bath adjusted to maintain a sediment temperature of 20 ± 1°C under a 16 hour light: 8 hour dark photoperiod. Temperature and dissolved oxygen (DO) concentrations in all replicates were measured daily. Conductivity, total ammonia, pH, alkalinity, and hardness were measured from composite samples collected from the replicate overlying water on day 0 and 10 to ensure consistency in water chemistry throughout. Statistical significance for animal survival was determined calculating differences from controls at the p = 0.05 level. Each endpoint was evaluated by first analyzing the data for normality and homogeneity of variance with Shapiro-Wilk's Test and/or Levene’s Test, respectively, before comparison of means. If the data were normally distributed and the variances were homogeneous, then analysis of variance (ANOVA) was utilized for the survival and weight data along with Dunnett's procedure for comparing the means. Survival data were also analyzed using Fisher’s Exact test. If the assumptions of normality or homogeneity of variance were not met, data were square-root transformed to allow the use of parametric procedures.

*Chironomid reproduction and survival*

Larval midges (*Chironomus riparius*) were obtained from in-house cultures and were 1^st^ instar larvae ranging from 1 to 3 days post-hatch. The test chambers were 450 mL glass jars containing approximately 100 mL of control or lake sediment. The overlying water was renewed using an automated system that provided approximately 3.7 volume additions per day. Each test chamber was fitted with a screened collar to allow water to drain from the chamber while retaining the test organisms. The animals were fed approximately 1.5 mL of a prepared invertebrate food (4 g L^-1^ fish flakes food suspension) daily. The number of live and dead animals in each test chamber was enumerated at test termination (day 10) by sieving the sediment through a 500 µm sieve. Animals were considered dead if they did not respond to a gentle physical stimulus. The criterion for test acceptability was mean survival in the controls of ≥ 70 %. Surviving larvae were dried at approximately 59-60 °C for 39 hours and weighed as a replicate group. Any pupae observed were included in the overall survival but were not included in the replicate weights. Each replicate was removed from the drying oven, placed into a dessicator to cool, and then weighed. After recording the dry weight, the sample was placed into a muffle furnace at 550 °C for two hours to ash. Samples were then removed from the furnace and allowed to cool in a dessicator and then weighed to the nearest 0.1 mg.

*Daphnia reproduction and survival*

Juvenile cladocerans (*Daphnia magna*) were obtained from in-house cultures. Animals utilized for the study were five days old at study initiation. Tests were conducted in 50 mL beakers each containing ~25 mL of overlying water and 5 mL of test sediment. Each control and lake sample was replicated 10 times. One *Daphnia magna* juvenile was added to each of 10 replicate beakers per control and site samples. All beakers were monitored daily for survival of *Daphnia* and for production of young. Overlying water in each replicate was renewed daily. Approximately 20 mL of the overlying water was renewed on a daily basis by removing the adult and temporarily placing it in a 50 mL beaker with fresh water and food. The remaining old solution was then removed along with any young present. Care was taken not to disturb the sediment in the bottom of the beaker. Old solutions were removed with a large bore glass pipette or a 60 mL syringe fitted with a piece of screen. A 2 L volume of renewal water was prepared daily by adding 13.3 mL of a concentrated live algal suspension and 13.3 mL of a daphnia supplement diet. At this concentration each beaker received approximately 0.1 mL of the algae and daphnia supplement on a daily basis. New solution volumes of ~20 mL and food were added to beakers and the adult daphniid returned to the beaker.

**Supplementary Information S3: Sediment core chronological and background geochemical information.**

Depth/age profiles; Bulk sediment accumulation rates; organic matter content (estimated as loss-on-ignition at 550 °C); ^137^Cs activity profiles; and iron (Fe) and manganese (Mn) profiles for each of the sediment cores analysed for trace metals.

**Supplementary Information S4: Trace metal data**

Trace metal concentration data for Hg, Pb, Cu, Ni, Zn and Cr. Note concentration units for Hg are ng g^-1^; the remainder are µg g^-1^. Vertical green and red lines are the Threshold Effects Concentration (TEC) and Probable Effects Concentration (PEC) for each element respectively.

**Supplementary Information S5: Persistent Organic Pollutants data**

Concentration data for ΣPCBs, HBCD, ΣPenta-BDEs, BDE-209 and ΣPBDEs. All units are ng g^-1^ dry weight. Vertical red lines are the Federal Environmental Quality Guidelines for ΣPenta-BDEs and BDE-209 (Environment Canada 2013). Equivalent FEQGs for ΣPCBs and HBCD far exceed the measured values so are not shown. No Guideline values for ΣPBDEs are available.

**References for Supplementary Information**

Abdallah, M. A., Harrad, S. & Covaci, A. (2009) Isotope Dilution Method for Determination of Polybrominated Diphenyl Ethers using Liquid Chromatography Coupled to Negative Ionization Atmospheric Pressure Photoionization Tandem Mass Spectrometry: Validation and Application to House Dust. *Analytical Chemistry, 81,* 7460–7467.

Clarke, A., (1969). The Story of Blackpool. S.R.Publishing Ltd. Wakefield.

Environment Canada (2013). Canadian Environmental Protection Act, 1999. Federal Environmental Quality Guidelines. Polybrominated Diphenyl Ethers (PBDEs). <https://www.ec.gc.ca/ese-ees/05DF7A37-60FF-403F-BB37-0CC697DBD9A3/FEQG_PBDE_EN.pdf> Accessed 31st August 2017.

Epstein, M. S., Diamondstone, B. I. & Gills, T. E. (1989) A new river sediment standard reference material. *Talanta, 36*, 141-150.

Harrad, S. & Hunter S. (2006) Concentrations of polybrominated diphenyl ethers in air and soil on a rural-urban transect across a major UK conurbation. *Environmental Science & Technology, 40*, 4548-53.

Harrad, S., Abdallah, M. A., Rose, N. L., Turner, S. D. & Davidson, T. A. (2009) Current-use brominated flame retardants in water, sediment, and fish from English lakes. *Environmental Science & Technology, 43,* 9077-9083.

Norberg King, T. J. (1999) Methods for measuring toxicity and bioaccumulation of sediment-associated contaminants and freshwater invertebrates. U.S. Environmental Protection Agency, Washington, DC, EPA/600/R-99/064. <https://cfpub.epa.gov/si/si_public_record_Report.cfm?dirEntryID=63220> Accessed 31^st^ August 2017.

Turner, S.D., Rose, N.L., Goldsmith, B., Harrad, S., Davidson, T. (2013). Opal Water Centre Monitoring Report 2008 – 2012. Open Air Laboratories, London. 204pp. <https://www.opalexplorenature.org/OPAL-water-report> Accessed 15^th^ September 2017.

US EPA (2002). Short-term methods for estimating the chronic toxicity of effluents and receiving waters to freshwater organisms (EPA 821/R-02/013) <https://www.epa.gov/sites/production/files/2015-08/documents/short-term-chronic-freshwater-wet-manual_2002.pdf> Accessed 31st August 2017.
